# Metagenomic next‐generation sequencing for the diagnosis of *Chlamydia psittaci* pneumonia

**DOI:** 10.1111/crj.13519

**Published:** 2022-06-20

**Authors:** Hu Li, Binwei Hao, Yongxue Wang, Dinghong Yu, Zhonghua Chen, Duanlin Du, Jian Xiong, Kang Li, Hengping Zhang, Xu Liu, Kai Liu, Fanrong Xiao, Xiaosan Cheng, Lingmei Huang

**Affiliations:** ^1^ Department of Imaging Yueyang Central Hospital Yueyang Hunan China; ^2^ Department of Pulmonary and Critical Care Medicine, Shanxi Bethune Hospital, Shanxi Academy of Medical Sciences, Tongji Shanxi Hospital Third Hospital of Shanxi Medical University Taiyuan China; ^3^ Tongji Hospital, Tongji Medical College Huazhong University of Science and Technology Wuhan China; ^4^ Department of Pulmonary and Critical Care Medicine Yueyang Central Hospital Yueyang Hunan China

**Keywords:** *Chlamydia psittaci*, clinical analysis, metagenomic next‐generation sequencing, pneumonia

## Abstract

The aim of this study was to investigate the clinical characteristics of *Chlamydia psittaci* pneumonia and evaluate the diagnostic value of Metagenomic Next‐Generation Sequencing (mNGS). A total of 44 patients diagnosed with *Chlamydia psittaci* pneumonia using mNGS were retrospectively analysed. The demographic and clinical features, laboratory data, imaging findings and clinical outcomes were collected. Results showed that 65.91% of the patients had a history of exposure to poultry or birds. All patients presented with fever. Apart from systemic and respiratory symptoms, some patients also presented with digestive and neurological symptoms. Respiratory failure was common among patients. The key laboratory tests were normal white blood cell counts, slightly elevated PCT, changes in levels of cardiac enzymes, liver enzymes and hyponatremia. Chest imaging revealed that most of the lesions contained patchy exudation or lobar consolidation of one lobe, especially in the lower lobe. Consolidation of both lungs was seen in critically ill patients. Although quinolones were effective in most patients, tetracyclines should be the first choice of treatment. The overall prognosis was good; however, patients who developed severe pneumonia had poor prognosis. The incidence o*f chlamydia psittaci* pneumonia may be underestimated due to the nonspecific clinical manifestations and lack of confirmatory testing methods. The use of mNGS has increased the number of patients diagnosed with *chlamydia psittaci* pneumonia. mNGS is an effective diagnostic method for *chlamydia psittaci* pneumonia.

## Graphical abstract

The clinical characteristics of *Chlamydia psittaci* pneumonia were analysed to inform clinical treatment. Early detection of mNGS is helpful for diagnosis and prognosis, and doxycycline is the preferred treatment.

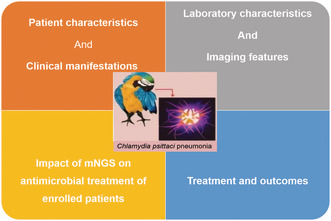

## 1 INTRODUCTION

*Psittacosis* is a human zoonotic disease caused by the bacterium *Chlamydophila psittaci*. *Psittacosis* is thought to be a sporadic cause of community‐acquired pneumonia (CAP), with an overall frequency of about 1%^1^; therefore, it tends to be overlooked by physicians. However, due to non‐specific clinical presentation, low awareness of the disease and limitations of diagnostic tests, psittacosis is highly underdiagnosed.^2^ In regions with frequent bird exposures, the prevalence could periodically rise up to 10–15%.^3^ Patients who do not undergo timely diagnosis and treatment can develop severe pneumonia and multiple organ failure, which may sometimes lead to death.^4^ Metagenomic next‐generation sequencing (mNGS), which can rapidly and precisely identify potential pathogens, has become the most promising approach for infectious disease diagnosis.^5^ With increasing applications of mNGS in diagnosis, the number of reported cases of *Chlamydia psittaci* pneumonia has gradually increased.^4^ Herein, we report 44 cases of *Chlamydia psittaci* pneumonia diagnosed by mNGS, describe their demographic and clinical features, laboratory data and imaging findings, clinical outcomes and the diagnostic performance of mNGS.

## 2 PATIENTS AND METHODS

### 2.1 Study design

This was a retrospective study involving 44 patients with *Chlamydia psittaci* pneumonia diagnosed using mNGS at Yueyang Central Hospital, a tertiary class A hospital in Yueyang City, Hunan Province, China, from 1 January 2019 to 30 November 2021. Clinical data of the patients, including demographic data, clinical manifestations, laboratory examination findings, imaging features, treatment approaches and outcomes, were collected from the electronic medical records and analysed.

### 2.2 Diagnostic criteria

Given that the detection methods for *Chlamydia psittaci*, such as culture, serum immunology and PCR, were not available in our hospital, the clinical diagnostic criteria for *Chlamydia psittaci* pneumonia in this study were as follows: (i) The enrolled patients met the diagnostic criteria for community‐acquired pneumonia (CAP)^6^; (ii) the presence of genetic fragments of *Chlamydia psittaci* in bronchoalveolar lavage fluid (BALF), blood or sputum samples revealed by mNGS; and (iii) conventional microbiological diagnostic methods, including blood, sputum and BALF cultures, were negative.

### 2.3 mNGS

mNGS tests were performed to identify the causative agent in patients with atypical pulmonary infection who showed poor response to empiric anti‐infection therapy or presented with severe symptoms on admission. According to the company's operating procedures (Beijing Genomics Institute, Beijing, China), the mNGS tests were performed as described below:

- Clinical samples (qualified sputum, BALF or blood) were collected in line with standard aseptic processing procedures.
- Immediately after collection, samples were transferred by an uninterrupted cold‐chain transportation to a genetic testing company.
- During genetic testing, DNA was extracted, DNA library was constructed by breaking the DNA fragment into 150 ~ 250 bp insert fragments, followed by terminal repair and adapter connection. PCR amplification was performed, and low‐quality, short fragments (length <35 bp) and human host fragments were removed. Finally, the remaining data were classified by aligning to four Microbial Genome Databases, including bacteria, viruses, fungi and parasites. The reference microbiome databases were downloaded from NCBI (ftp://ftp.ncbi.nlm.nih.gov/genomes/).^7^, ^8^

### 2.4 Statistical analysis

Data analysis was performed using the SPSS 25.0 software. Quantitative data with normal distribution and homogeneity of variance are presented as mean ± standard deviation (SD), while the remaining quantitative data are presented as medians (25th, 75th percentiles). Categorical data are expressed as counts and percentages.

## 3 RESULTS

### 3.1 Patient characteristics

Between 2019 and 2021, a total of 44 *Chlamydia psittaci* pneumonia cases (20 cases were male, while 24 were female, aged 56.86 ± 8.57 years) were diagnosed by mNGS. Twenty‐nine patients (65.91%) had an exposure history, including 23 cases of exposure to poultry (chicken and ducks) and 6 cases to pigeons. The remaining 15 patients denied any direct exposure history. Twenty‐six patients had underlying diseases **(**Table 1 **)**.

**TABLE 1:** Demographic characteristics and clinical manifestations

Patient characteristics	Total (*n* = 44), *n* (%)	Value
Demographics		
Age (years)		56.86 ± 8.57
Female	24 (54.55%)	
History of contact	29 (65.91%)	
Underlying disease	26 (59.09%)	
Onset in Autumn and winter	31 (70.45%)	
Time from onset to hospitalization (days)		5.5 (3.75–7)
Time from specimen collection to mNGS results (days)		2.23 ± 0.73
Time from hospitalization to diagnosis (days)		5.17 ± 2.31
Clinical manifestations		
Fever > 38.5°C	44 (100%)	39.53 ± 0.56
Relatively slow pulse	27 (61.36%)	
Chills	29 (65.91%)	
Weakness	23 (52.27%)	
Anorexia	15 (34.09%)	
Myalgia	4 (9.09%)	
Cough	36 (81.82%)	
Dyspnea	20 (45.45%)	
Gastrointestinal symptom	16 (36.36%)	
Neurological symptom	22 (50.0%)	
Respiratory failure	16 (36.36%)	
Shock	4 (9.09%)	
Severe pneumonia	6 (13.64%)	

### 3.2 Clinical manifestations

Five patients were admitted in 2019, 19 in 2020 and 20 in 2021. Onset seasons were mainly autumn and winter (September to February), with 31 cases admitted, while only 13 cases were admitted in spring and summer (March to August). The time from onset to admission was 5.5 (3.75–7) days, whereas that from specimen submission to mNGS results reporting was 2.23 ± 0.73 days, and time from hospitalization to diagnosis was 5.17 ± 2.31 days. All patients had fever. A relatively slow pulse was found in 27 cases. Generalized symptoms included chills, weakness, anorexia and myalgia. The primary respiratory symptoms were cough and dyspnea. Some patients presented with digestive symptoms, including nausea, vomiting and diarrhoea. In addition, some patients experienced headaches and had disturbances of consciousness. Sixteen patients developed respiratory failure, four patients went into septic shock and six patients met the diagnostic criteria for severe pneumonia (Table 1).

### 3.3 Laboratory characteristics

Upon admission, peripheral white blood cell (WBC) counts were 7.92 ± 3.06 (×10^9^/L). The WBC counts for a majority of the patients were normal. Neutrophil percentage was 83.87 ± 7.17. C‐reactive protein (CRP) levels were markedly increased, with a mean of 123.05 mg/L. Procalcitonin (PCT) was slightly increased, with a mean of 0.46 ng/ml. All detected patients had elevated Erythrocyte sedimentation rate (ESR), with a mean of 76 mm/h. Of the evaluated 38 cases, 47.37% and 86.84% had elevated lactate dehydrogenase (LDH) and myoglobin (MYO) levels. In addition, 50.0% of the patients exhibited elevated alanine aminotransferase (ALT) levels, while 63.64% of the patients had elevated aspartate aminotransferase (AST) levels. Hyponatremia was the most common type of electrolyte disorder. Other abnormalities included elevated D‐Dimer (DD_2_) and fibrinogen levels as well as elevated IL‐6 (Table 2).

**TABLE 2:** Laboratory findings and radiologic features

Characteristics	Patients, *n* (%)	Value
Laboratory findings		
Elevated WBC (3.5–10 × 10^9^/L)	9/44 (20.45%)	7.92 ± 3.06
Elevated NEU% (40–75%)	38/44 (86.37%)	83.87 ± 7.17
Elevated CRP (0–8 mg/L)	42/42 (100%)	123.05 (105.8–133.05)
Elevated PCT (0–0.05 ng/mL)	44/44 (100%)	0.46 (0.21–1.49)
Elevated ESR (0–15 mm/h)	37/37 (100%)	76(67–104.5)
Elevated LDH (120–250 U/L)	18/38 (47.37%)	261.55 (156.75–392.6)
Elevated MYO (0–85 U/L)	33/38 (86.84%)	261.75 (153.08–523.58)
Elevated ALT (9–50 U/L)	22/44 (50.0%)	69.04 ± 50.10
Elevated AST (15–40 U/L)	28/44 (63.64%)	57.79 (34.58–95.70)
Hyponatremia (137–147 mmol/L)	32/44 (72.73%)	131.11 ± 4.88
Decreased PaO2 (>60 mmHg)	16/35 (45.71%)	56.5 (52.0–71.5)
Elevated D‐Dimer (0–500 ug/L)	37/42 (88.10%)	1555 (690–2900)
Elevated fibrinogen (2–4 g/L)	39/39 (100%)	6.83 ± 1.39
Elevated IL‐6 (0–5.4 pg/mL)	20/20 (100%)	130.38 (49.95–248.18)
Imaging		
Patchy shadows	44/44 (100.0%)	
Consolidation	25/44 (56.82%)	
Single lung involved	31/44 (70.45%)	
Lesion began in the lower lobe	29/44 (65.91%)	
Pleural effusion	27/44 (61.36%)	

Abbreviations: ALT, alanine aminotransferase; AST, aspartate aminotransferase; CRP, C‐reactive protein; ESR, erythrocyte sedimentation rate; *IL*‐6, interleukin‐6; LDH, lactate dehydrogenase; LYM, lymphocyte; MYO, myoglobin; NEU, neutrophils; PaO_2_, partial pressure of arterial oxygen; PCT, procalcitonin; WBC, white blood cells; 1 mmHg = 0.133 kPa.

### 3.4 Imaging features

All the patients were subjected to lung CT examination upon admission. They all showed patchy shadows. Segmental or lobar consolidations were presented in 56.82% of the patients, while 4.55% exhibited ground glass shadows. There were 31 cases with unilateral lesions, and 13 cases had bilateral lesions. Lesions were most common in the lower lobes (29 cases). As the disease progressed, multiple lobes were involved in 19 cases. There were 27 cases of pleural effusions, all of which were small effusions (Table 2).

### 3.5 mNGS

Diagnostic confirmation was performed by sequencing *Chlamydia psittaci* from BALF, peripheral blood or qualified sputum samples using mNGS. Among them, 35 patients were confirmed by sole BALF samples, 7 by sole blood, 1 by sole sputum and 1 by both qualified sputum and blood samples. The mNGS sequence number for *Chlamydia psittaci* ranged from 1 to 2653 in BALF, 203 to 1057 in sputum and from 3 to 425 in blood (Table 3). In addition to *Chlamydia psittaci*, most of the other pathogens detected by mNGS were background bacteria or colonized bacteria, which had no clinical significance (Table S1).

**TABLE 3:** Detection of chlamydia psittaci by mNGS

mNGS specimen	Total (*n* = 44), *n* (%)	Sequence number
BALF Only	35 (79.55%)	1–2653
Blood Only	7 (15.91%)	3–32
Sputum Only	1 (2.27%)	203
Sputum and Blood	1 (2.27%)	Sputum (1057), Blood (425)

Abbreviation: BALF, bronchoalveolar lavage fluid.

### 3.6 Impact of mNGS on antimicrobial treatment of enrolled patients

On admission, all patients received empirical antimicrobial treatment comprising quinolones, β‐lactamases, glycopeptides and antivirals. Up to 77.27% of the patients received combination antimicrobial therapy. Thirty‐five patients were initially treated with quinolones, and 11 patients with doxycycline before mNGS. According to mNGS results, 79.55% of patients changed the type of antimicrobial drug, 25% had one or two antimicrobial agents removed and 63.64% received down‐stair antibiotic treatment. Moreover, 63.64% of patients did not receive doxycycline treatment before mNGS testing. The antibiotics were not changed in nine patients (Table 4).

**TABLE 4:** Impact of mNGS on antimicrobial treatment of enrolled patients

Observation index	Total (*n* = 44), *n* (%)
Initial empiric treatment before mNGS	
Combination of antimicrobial drugs	34 (77.27%)
Use of doxycycline	11 (25.0%)
Modifications based on mNGS	35 (79.55%)
Remove 1 antimicrobial drug	5 (11.36%)
Remove 2 antimicrobial drugs	6 (13.64%)
De‐escalation therapy of antibiotics	28 (63.64%)
Add doxycycline	28 (63.64%)
Add quinolones	7 (15.91%)
Add azithromycin	1 (2.27%)
No change	9 (20.45%)

### 3.7 Treatment and outcomes

With regards to efficacies, 71.43% of the patients administered with quinolones only improved, while 10 patients showed no improvement until doxycycline was added. The mean antipyretic time was 4 (3–5) days. Forty patients required oxygen support, including 35 with nasal catheters, 2 with non‐invasive ventilation, 3 with high‐flow nasal cannula therapy and 4 with invasive mechanical ventilation, including 1 with extracorporeal membrane oxygenation (ECMO). Complications included deep venous thrombosis in three patients, shock in four patients and gastrointestinal bleeding in one patient. Finally, 42 patients were cured and discharged; one patient was unrecovered, and one patient died. The time from admission to discharge was 10 (8–14) days (Table S2).

## 4 DISCUSSION

*Chlamydophila psittaci* is an obligate intracellular Gram‐negative bacterium of the *Chlamydiaceae* family.^9^ Compared to other *Chlamydophila* species, *Chlamydophila psittaci* is more pathogenic, and it tends to cause severe inflammatory responses and high mortality. Wu et al.^10^ reported that *Chlamydophila psittaci* accounts for about 7.3% of severe CAP in immunocompetent patients. Given the large number of CAP patients, *Chlamydophila psittaci* should be a pathogen that physicians take seriously.

*Psittacines* (parrot‐type birds) are the main carriers of *Chlamydia psittaci*. In addition, pigeons, turkeys, ducks and chickens have been implicated as sources of infections.^11^ Moreover, mammals such as horses,^12^ cats and dogs^13^ are possible sources. Humans can contract *psittacosis* after contact or inhalation of aerosolized bacteria from infected secretions, feathers or droppings.^9^ In this study, most of the patients had a contact history with poultry or pigeons; however, 34.10% of the patients denied any contact history. Detailed information is needed when assessing patient history. Human‐to‐human transmission has been reported,^14^ but no case was found in this study. A study found that parrots were the most common sources of infections in women while pigeons in men.^15^ In our study, infection sources were mainly poultry and pigeons, and there were no significant differences in infection sources among genders. The finding is consistent with the results of a meta‐analysis,^11^ suggesting that when tracing disease origin, focus should be on parrots, poultry and other birds.

Most cases occurred in autumn and winter, similar to a study in Australia that found a high incidence of *psittacosis* during the cold season (March–May, Australia in the southern hemisphere).^3^ However, a study from the Netherlands, another northern hemisphere country, found that *psittacosis* is most common in spring and summer.^16^ Therefore, the correlation between *psittacosis* onset and season should be evaluated further.

The incubation period ranges from 7 to 40 days, with symptoms ranging in severity, from asymptomatic to life‐threatening multi‐organ disease.^17^ In general, clinical presentation of *Chlamydia Psittaci* pneumonia is non‐specific.^18^ The main clinical manifestations are fever, chills, muscle aches, fatigue, cough, headache, diarrhea, among other symptoms.^17^ Severe cases may involve respiratory failure, psychiatric symptoms, circulatory failure, acute renal failure or even death.^4^, ^17^ According to our study, the clinical characteristics of *Chlamydia Psittaci* pneumonia were as follows: (i) All patients presented with fever, with high fever (>39°C) being more common; (ii) most patients presented with coughs, expectoration, accompanied by fatigue, dizziness, headaches, body muscle aches and other flu‐like symptoms; (iii) extrapulmonary manifestations were high and varied, and they included digestive symptoms and neurological symptoms, some of which preceded or were concomitant fever; (iv) most of the patients showed relatively slow pulse rates, which was not synchronized with the increase in pulse when patients had high fevers, and the two were separated on the body temperature list; (v) patients in this group could have a rapid disease progression and a high rate of severe cases; some patients may develop respiratory failure and progress into severe pneumonia; and (vi) some patients gradually progressed into septic shock, which necessitated the provision of organ support therapy.

In laboratory tests, some findings were as previously reported in other studies,^3^, ^19^ including (i) normal WBC counts, high neutral ratio and low lymphocyte ratios; (ii) inflammation indices, such as CRP and ESR, were significantly elevated, while PCT was only slightly elevated; and (iii) increased myocardial enzyme (LDH and MYO), increased liver enzyme (ALT and ALT) and hyponatremia were detected in some patients. In addition, some of our findings that have rarely been reported were as follows: (i) Among inflammatory factors, IL‐6 was significantly elevated. Since IL‐6 is a highly sensitive and specific inflammatory indicator, elevated IL‐6 levels imply severe inflammatory responses in *Chlamydia psittaci* pneumonia patients, which can aid in its effective evasion and adaptation.^20^ (ii) In terms of coagulation, fibrinogen and D‐dimer levels were significantly elevated. Since elevated D‐dimer and fibrinogen levels are associated with activation of the coagulation system and closely related to the severity and prognosis of pneumonia patients,^21^ the appropriate anticoagulant therapy may be needed for the patients with a hypercoagulable state.

The main changes in lung imaging of *Chlamydia psittaci* pneumonia are exudation and consolidation of varying degrees. The most common manifestations of lung CT are unilateral and single‐lobed consolidation, followed by unilateral and bilateral multilobed consolidation. The lower lobes were frequently involved, in tandem with a previous report.^19^ As the disease progressed, multiple lobes were involved. In addition, a small amount of pleural effusion occurred in some patients, and lymphadenopathy was rare.

Serious conditions can occur if appropriate medical treatments are delayed.^4^ Microbiologic diagnosis of *psittacosis* is the ‘rate‐limiting’ step for timely treatment. Currently, cultures, serology and PCR are the conventional diagnostic methods for *psittacosis*.^2^ Culture is time‐consuming and requires biosafety level 3 facilities, thus not routinely performed in most laboratories.^22^ Micro‐immunofluorescence test (MIF) is considered to be the gold serological test method with high sensitivity.^22^ But, since the test conditions for MIF are highly variable, this may present difficulties in interpreting the results,^23^ and false positive results may occur due to possible cross‐reactions with other *chlamydia*.^24^ PCR is a rapid and accurate detection method with high sensitivity and specificity in acute phases.^2^, ^22^ Unfortunately, due to technical difficulties and relatively expensive reagents, this approach is not widely applied and has only been carried out in suspected cases.^2^ mNGS has become a promising and universal method for fast and accurate pathogen identification.^25^ mNGS can detect multiple pathogens at the same time, including atypical pathogens, viruses, fungi and other pathogens that are difficult to culture, sparing the need to identify suspected pathogens in advance. In our study, patients were diagnosed with *psittacosis* through mNGS of BALF, blood or sputum samples. The sequence number was highest in BALF, followed by qualified sputum and lowest in blood samples. Therefore, BALF should be the first‐choice sample for mNGS.^26^ Nine people who either declined or could not tolerate bronchoscope examination were diagnosed using mNGS of blood or sputum samples, indicating that blood or qualified sputum samples are good alternatives when BALF is not available.

In this study, mNGS was performed for patients with atypical pneumonia who had poor response to empirical treatment or were in critical condition at admission. The results of mNGS were obtained 2.23 ± 0.73 days after specimen submission. Conventional cultures in our hospital take 3–5 days, and majority of the cultures are negative. Therefore, the mNGS method provides an accurate and rapid detection of infectious pathogens which overcomes the limitations of conventional cultures. Since 2019, the number of patients with *chlamydia psittaci* pneumonia has been on the rise due to increased awareness of the disease among clinicians and a decrease in missed diagnoses owing to the use of mNGS. This suggests that this disease was significantly underdiagnosed and misdiagnosed and its true incidence requires further epidemiological investigation.

Overuse of antimicrobial drugs can lead to drug resistance and waste of medical resource. In this study, 77.27% patients received more than two antibiotics at admission, and nearly 50% of patients received carbapenems. Based on the results of mNGS, antimicrobial drugs adjustment was performed for 79.55% patients, either as antibiotic downgrading or discontinuation of combinations of broad‐spectrum doxycycline or continuation of quinolones. Therefore, adoption of mNGS reduces unnecessary use of antibiotics (such as carbapenems) and, thus, the risk of bacterial resistance. Moreover, 63.64% of patients did not receive doxycycline treatment before mNGS was performed, suggesting that mNGS can accurately diagnose the disease which is essential for appropriate therapy. Of note, 11 patients admitted after October 2020 received empirical doxycycline before mNGS testing, which may be due to the increase in the number of confirmed patients with *Chlamydia psittaci* pneumonia and awareness of the disease among clinicians.

However, since mNGS can detect many pathogens simultaneously and there is a lack of a widely accepted threshold value, one tricky challenge in interpreting mNGS results is determining whether the detected microorganisms are pathogenic or normal flora,^27^ which requires clinicians to make judgements based on clinical situations. As *chlamydia psittaci* does not exist in normal human samples; therefore, although the sequence number for some patients was very low in our study, it still has a diagnostic value. Additionally, the high cost of mNGS, which costs about 400–600 dollars per test, limits its clinical applications.

Drugs used to treat *Chlamydia psitsiti* pneumonia include tetracyclines such as doxycycline or minocycline, macrolides and quinolones.^28^ Since fluoroquinolones are the most commonly used antibiotics for CAP, the number of patients initially treated with fluoroquinolones was as high as 75.5%. Most patients showed good treatment outcome, but 28.57% patients showed poor response to this treatment. The high failure rate of quinolones in patients with *Chlamydia psitsiti* pneumonia is consistent with results from previous studies.^17^ Therefore, quinolones are not recommended as the preferred treatment for *Chlamydia psittaci* pneumonia. The efficacy of macrolides was not being determined in this study because only one patient received azithromycin combined with doxycycline and moxifloxacin. Tetracyclines are recommended as the first‐line treatment for *Chlamydia psittaci* pneumonia.^28^ Indeed, in this study, patients initially treated with doxycycline on admission responded quickly to treatment as indicated by decreased body temperature, improvement of cough, fatigue, poor appetite, shortness of breath and absorption of pulmonary lesions based on imaging results. Doxycycline was effective in patients who showed poor response to quinolones. However, it should be noted that with the widespread use of tetracycline drugs in clinical and agricultural settings, tetracycline resistance is on the rise. Possible mechanisms of resistance include ribosomal protection, efflux or enzyme inactivation.^29^ Several studies have investigated strategies to overcome resistance to tetracycline. For example, a new generation of tetracycline was developed which can inhibit ribosomal protection and overcome drug resistance.^30^ In addition, resistance may be overcome through a combination strategy of new tetracycline/tetracycline destructase inhibitor to overcome enzyme inactivation.^29^ Therefore, tetracycline combined with macrolides or quinolones may be effective in patients with severe life‐threatening tetracycline resistance.

Generally, *Chlamydia psittaci* pneumonia has good prognostic outcomes; however, if patients do not receive timely and correct treatment, the mortality rate can rise to 10–20%.^28^ In this study, 36.36% of the patients developed respiratory failure, and up to 13.64% of patients progressed to severe pneumonia. Among them, four patients were provided with invasive ventilator‐assisted ventilation, and one patient also received ECMO adjuvant therapy. Overall, the prognosis was good, with 42 patients improved and discharged. However, one patient's condition did not improve after receiving invasive assisted ventilation for 29 days and discharged. Another patient presented with severe pneumonia developed shock, massive gastrointestinal bleeding and venous thrombosis of lower limbs successively and died of sudden respiratory cardiac arrest, possibly due to fatal pulmonary embolism, which resulting from failure to manage bleeding and clotting balance.

Our study has some limitations. First, this was a small retrospective study. Second, some laboratory tests such as blood gas and cytokine analyses were not performed for all patients. Third, in most patients, mNGS was only performed after failure of empirical treatment, which may have affected the results. Fourth, since serology, culture and PCR were unavailable in our hospitals, they were not used to confirm diagnosis. In addition, considering that samples were sent for testing in commercial laboratories, the transportation process may have affected the experimental data.

In conclusion, the number of patients with *Chlamydia psittaci* pneumonia has been on the rise, and clinical awareness of the disease has also increased, which is attributed to the increased adoption of mNGS testing. The use of mNGS improves the treatment and hence prognosis of the disease.

## CONFLICT OF INTEREST

The authors have declared that they have no competing interest.

## ETHICS STATEMENT

This study was conducted according to the principles of the Declaration of Helsinki and approved by the Ethical Committee of Yueyang Central Hospital. Meanwhile, due to the retrospective nature of the study, the Ethical Committee of Yueyang Central Hospital waived the need for patients' approval and informed consents. All research data were anonymously analysed.

## AUTHOR CONTRIBUTIONS

Lingmei Huang and Hu Li wrote the original draft. Duanlin Du, Jian Xiong, Kang Li, Hengping Zhang, Kai Liu, Xu Liu and Fanrong Xiao provided some of the data. Yongxue Wang, Dinghong Yu and Zhonghua Chen undertook validation, writing, review and editing. Binwei Hao and Sanxiao Cheng undertook review and editing. All authors read and approved the submitted version.

## Supporting information


**Table S1.** The detailed information of mNGS results and reads

**Table S2.** Treatment and clinical outcomes of enrolled patients

## Data Availability

No additional data are available.
