# Supplementary material for: Metagenomic next‐generation sequencing for the diagnosis of Chlamydia psittaci pneumonia
Source: Clin Respir J. 2022 Jun 20;16(7):513–21. doi: 10.1111/crj.13519 (PMC9329019; doi:10.1111/crj.13519)
Supplement: Supplementary file 1 — Table S1. The detailed information of mNGS results and reads [file CRJ-16-513-s002.docx]

| **Table 1** The detailed information of mNGS results and reads | | |
| --- | --- | --- |
| Patient | Specimen for mNGS | mNGS results and reads |
| 1 | BALF | *Chlamydia psittaci (24)* |
| 2 | BALF | *Chlamydia psittaci (14)* |
| 3 | BALF | *Chlamydia psittaci (155), Grevenny actinomycetes (27), dental actinomycetes (5), Dialisteria turbidis (32)* |
| 4 | BALF | *Chlamydia psittaci (35)* |
| 5 | BALF | *Chlamydia psittaci (364), Dental actinomycetes (8)* |
| 6 | BALF | *Chlamydia psittaci (16)* |
| 7 | BALF | *Chlamydia psittaci (68)* |
| 8 | BALF | *Chlamydia psittaci (58)* |
| 9 | Blood | *Chlamydia psittaci (3)* |
| 10 | BALF | *Chlamydia psittaci (45), Aspergillus Niger (1)* |
| 11 | BALF | *Chlamydia psittaci (23)* |
| 12 | Blood | *Chlamydia psittaci (15)* |
| 13 | BALF | *Chlamydia psittaci (43)* |
| 14 | Sputum | *Chlamydia psittaci (203), Chlamydia abortus (21)* |
| 15 | BALF | *Chlamydia psittaci (2653)* |
| 16 | BALF | *Chlamydia psittaci (5)* |
| 17 | BALF | *Chlamydia psittaci (1), Herpes simplex virus (119)* |
| 18 | Blood | *Chlamydia psittaci (16), Human herpesvirus type 5(15)* |
| 19 | BALF | *Chlamydia psittaci (130)* |
| 20 | BALF | *Chlamydia psittaci (564)* |
| 21 | BALF | *Chlamydia psittaci (219)* |
| 22 | BALF | *Chlamydia psittaci (812), Corynebacterium glucuronid (4)* |
| 23 | BALF | *Chlamydia psittaci (32)* |
| 24 | BALF | *Chlamydia psittaci (15)* |
| 25 | BALF | *Chlamydia psittaci (76)* |
| 26 | Blood | *Chlamydia psittaci (7)* |
| 27 | BALF | *Chlamydia psittaci (28)* |
| 28 | BALF | *Chlamydia psittaci (178), Pseudomonas maltophilia (271), Candida albicans (71)* |
| 29 | BALF | *Chlamydia psittaci (56)* |
| 30 | BALF | *Chlamydia psittaci (33)* |
| 31 | BALF | *Chlamydia psittaci (74)* |
| 32 | BALF | *Chlamydia psittaci (48), Haemophilus arainfluenzae (32)* |
| 33 | BALF | *Chlamydia psittaci (34), Psedumonas aeruginosa (132), Streptococcus pneumoniae (67), Stenotrophomonas maltophilia (454), Streptococcus bradyces (123)* |
| 34 | Blood | *Chlamydia psittaci (11), Propionic acid bacillus (34), Common coccus epidemids (542)* |
| 35 | BALF | *Chlamydia psittaci (17)* |
| 36 | Blood | *Chlamydia psittaci (5)* |
| 37 | BALF | *Chlamydia psittaci (3), Stenotrophomonas maltophilia (168)* |
| 38 | BALF | *Chlamydia psittaci (201)* |
| 39 | BALF | *Chlamydia psittaci (152),Aspergillus fumigatus (2),Haemophilus influenzae (4）* |
| 40 | BALF | *Chlamydia psittaci (263), Denitrogenia aureus (583)* |
| 41 | BALF | *Chlamydia psittaci (41)，Candida albicans (15)* |
| 42 | Blood | *Chlamydia psittaci (32)* |
| 43 | Blood | *Chlamydia psittaci (425)* |
| 43 | Sputum | *Chlamydia psittaci (1057)* |
| 44 | BALF | *Chlamydia psittaci (65), Burkolderia onion (1544), aspergillus flavus (45)* |
