# Supplementary material for: Metagenomic next‐generation sequencing for the diagnosis of Chlamydia psittaci pneumonia
Source: Clin Respir J. 2022 Jun 20;16(7):513–21. doi: 10.1111/crj.13519 (PMC9329019; doi:10.1111/crj.13519)
Supplement: Supplementary file 2 — Table S2. Treatment and clinical outcomes of enrolled patients [file CRJ-16-513-s001.docx]

**Table 2 Treatment and clinical outcomes of enrolled patients**

| **Observation index** | **Patients, n (%)** | **Value** |
| --- | --- | --- |
| **Efficiency of quinolones** | 25/35 (71.43%) |  |
| **Antipyretic time (days)** |  |  |
| Overall |  | 4.0 (3.0-5.1) |
| Quinolones  Doxycycline |  | 4.0 ± 0.33  3.79 ± 1.91 |
| Quinolones and Doxycycline |  | 3.47 ± 0.83 |
| **Respiratory support** |  |  |
| No oxygen support | 4/44 (9.09%) |  |
| Nasal tube oxygen | 35/44 (79.55%) |  |
| Non-invasive ventilation | 2/44 (4.55%) |  |
| High-flow nasal cannula | 3/44 (6.82%) |  |
| Invasive mechanical ventilation | 4/44 (9.09%) |  |
| ECMO | 1/44 (2.27%) |  |
| **Complications** |  |  |
| Deep venous thrombosis | 3/44 (6.82%) |  |
| Shock | 4/44 (9.09%) |  |
| Gastrointestinal bleeding | 1/44 (2.27%） |  |
| **Outcome** |  |  |
| Improved | 42/44 (95.45%) |  |
| unrecovered | 1/44 (2.27%) |  |
| Dead | 1/44 (2.27%） |  |
| **The hospitalization time (days)** |  | 10 (8-14） |

***ECMO:*** extracorporeal membrane oxygenation
